# Modification of conservative treatment of heterotopic cervical pregnancy by Foley catheter balloon fixation with cerclage sutures at the level of the external cervical os: a case report

**DOI:** 10.1186/1752-1947-4-212

**Published:** 2010-07-16

**Authors:** Tomislav Hafner, Ivana Erceg Ivkosic, Alan Serman, Renato Bauman, Boris Ujevic, Sanja Vujisic, Daria Hafner, Berivoj Miskovic

**Affiliations:** 1 Department of Obstetrics and Gynaecology, Clinical Hospital Sveti Duh, Zagreb, Croatia

## Abstract

**Introduction:**

Conservative treatment of a heterotopic cervical pregnancy was performed with a modification of the fixation of a Foley catheter at the level of the external cervical os, followed by the ligature of the descending cervical branches of the uterine arteries and systemic methotrexate application.

**Case presentation:**

A 34-year-old Caucasian woman was diagnosed with double gestation after 6 weeks of *in vitro *fertilization treatment. A gynecological examination and color Doppler ultrasound scan revealed intra-uterine and cervical gestational sacs both containing live fetuses. A Foley catheter balloon was inserted into the cervical canal, inflated and fixed by a cerclage suture at the level of the external cervical os, followed by ligation of the descending cervical branches of the uterine arteries. Systemic methotrexate was applied. Three days after removal of the Foley catheter, an evacuation of the intra-uterine gestational sac was performed. Hemorrhage from the implantation site was controlled immediately and a pregnancy termination was successfully performed. The procedure was uneventful and our patient was discharged with a preserved uterus.

**Conclusions:**

Conservative treatment of cervical pregnancy using a Foley catheter balloon is more efficacious if the Foley catheter balloon is attached in the correct position with a cerclage suture at the level of the external os, followed by ligation of the descending cervical branches of the uterine arteries, thereby exerting maximal pressure on the bleeding vessels.

## Introduction

Heterotopic pregnancy (HP) is a multiple pregnancy in which one or more intra-uterine pregnancies coexist with ectopic ones. It is a very rare entity. The incidence of HP has increased as the use of assisted reproductive technologies (ART) has become more widespread [1]. The first report of an ectopic pregnancy following *in vitro *fertilization-embryo transfer (IVF-ET) was published in 1976, and subsequently cases have been reported at an increasing rate with an incidence of 0.25% to 1.4% of all pregnancies [2]. Cervical ectopic pregnancy is a rare form of ectopic pregnancy and it accounts for approximately 0.15% of all ectopic pregnancies. A literature review demonstrated that the occurrence of a cervical pregnancy combined with an intra-uterine pregnancy, heterotopic cervical pregnancy, is quite an exceptional event and only a few case reports are described. In undiagnosed cervical pregnancies and those not treated early enough, catastrophic hemorrhage can occur often resulting in the necessity of a life-saving hysterectomy. However, since 1953 no maternal death has been reported in the literature as a consequence of cervical pregnancy. The etiology of cervical ectopic pregnancy is still unclear. Risk factors for cervical pregnancy include uterocervical anomalies, cervical stenosis, use of an intra-uterine device, previous abortions, uterine curettage, a previous Cesarean section, and infertility with tubal pathology and IVF treatment with ET. Early diagnosis is available with a Doppler ultrasound scan or MRI. In 1978 the first sonographic diagnosis of a cervical pregnancy was reported. The diagnosis should be accompanied by color Doppler to identify the peritrophoblastic flow [3]. The therapeutical approach can be radical or conservative. When radical, a hysterectomy is usually performed when hemorrhage occurs as an emergency situation. Conservative treatment can be surgical or medical, and allows for subsequent gestations. Most cervical ectopic pregnancies can be safely managed in a minimally invasive manner [4].

A local tamponade with an inflated Foley catheter balloon was first reported in 1983 [5]. Interestingly, only sporadic case reports describe application of an inflated Foley catheter balloon as a local tamponade in the face of a hemorrhagic bed of a cervical pregnancy, even though the method is simple and safe.

Our report describes a case of a heterotopic cervical pregnancy treated conservatively by a Foley catheter balloon inserted within the cervical canal, inflated and fixed by cerclage suture at the level of the external cervical os, followed by ligation of the descending cervical branches of the uterine arteries and systemic methotrexate application. A review of the literature is also presented.

## Case presentation

A 34-year-old Caucasian woman, gravida 4, para 0, with a history of one spontaneous abortion in early pregnancy and two extrauterine tubal pregnancies treated by laparoscopic left and right salpingectomy, self-referred to our department due to painless vaginal bleeding. She was six weeks pregnant after a successful IVF-ET program, according to our institution's protocol. Two blastocysts were transferred. Her gynecological findings on admission were as follows: smaller vaginal blood coagulum, mild fresh uterine hemorrhage, livid cervix, the external cervical ostium was closed and our patient had a six-week-sized uterus with unremarkable adnexal findings. Our patient's abdomen was soft and not tender. The first color Doppler transvaginal ultrasound scan revealed an intra-uterine gestational sac and live embryo with a crown-rump length (CRL) of 4 mm. Physical examination of our patient, performed at admission, showed normal temperature, blood pressure and heart rate. The established diagnosis was a threatened abortion. A few days later our patient experienced heavy vaginal bleeding with clots. She was re-examined and a color Doppler transvaginal ultrasound revealed another gestational sac with a live embryo in the cervical region. The CRL was 3.8 m. Her estimated blood loss was more than 600 mL in 24 hours reducing her hemoglobin level from 131 g/L to 109 g/L. Liver and renal function tests were within normal limits. Four units of blood were cross-matched to her blood. Her serum β-human chorionic gonadotropin (β-HCG) concentration was 217,000 IU/L. Our patient was cautioned that conservative management could result in a hysterectomy and her written consent was obtained before the procedure. Under general anesthesia a Foley catheter balloon was inserted within the cervical canal, controlled by ultrasound guidance, inflated and fixed by cerclage sutures at the level of the external cervical os, followed by ligation of the descending cervical branches of the uterine arteries. Hemorrhage from the implantation site was controlled immediately. Our patient was counseled again regarding the continuation of her intra-uterine pregnancy, but she decided to start with methotrexate treatment being fully aware that she would lose the other embryo. Systemic methotrexate application was started at 1.5 mg/kg with folic acid because of the adverse effects of this therapy. The Foley catheter was removed 3 days later and evacuation of the intra-uterine gestational sac was performed. Our patient recovered well and her progress was observed by regular transvaginal ultrasound scans and measurements of β-HCG concentrations. A transvaginal color Doppler examination showed a 3 mm thin hyperechogenic endometrium and anechogenic zone in the cervical canal (28 × 12 mm) with no detectable blood flow. At six months after the procedure, our patient resumed her regular menstrual cycles and one year later she underwent another IVF-ET procedure with an unknown outcome.

## Discussion

A modification of a conservative treatment for heterotopic cervical pregnancy by fixation of a Foley catheter balloon with a cerclage suture at the level of the external cervical os is an approach for using a Foley catheter balloon tamponade as a safe, simple and effective method without adverse effects, since it was used previously [6] and then inexplicably abandoned for some time. Hysterectomy can be avoided by early diagnosis and by an adequate tamponing technique to control hemorrhage. The use of a cervical canal tamponade with a Foley catheter balloon led to reliable hemostasis in 92.3% of the cases in which this method was used. Conservative approaches in heterotopic cervical pregnancies are, however, possible for up to 10 to 12 weeks of pregnancy, as with advanced pregnancy the trophoblastic tissue infiltrates deeply into the cervical wall. For the second or third trimester, or for uncontrolled bleeding, hysterectomy is recommended, although a report of conservative management of a second-trimester cervical ectopic pregnancy with placenta percreta has been published [7].

All reports currently published concerning heterotopic cervical pregnancy treated with a Foley catheter balloon differ from our approach. Fylstra and Coffey [8], for example, placed the cerclage suture high on the cervix and followed with a cervical canal tamponade with a Foley catheter balloon. De La Vega [9], for the treatment of a spontaneous cervical pregnancy after eight weeks of amenorrhea, used a combined cervical cerclage, carboprost, curettage and balloon tamponade after curettage.

Currently there are no specific recommendations for the best treatment of heterotopic (cervical) pregnancy and there is no universally accepted treatment modality. Methotrexate, administered either systemically or locally, has been reported to be an effective therapy [10], but the incidence of cervical pregnancy is rare and thus it is impractical to perform a prospective, randomized, controlled study to evaluate the efficacy of the different therapeutic regimens of methotrexate treatment, as is the case for all other treatments as well. Methotrexate is an agonist of folic acid that participates in DNA synthesis and has the capacity to stop proliferative cell activity. Transvaginal ultrasound-guided intra-amniotic instillation of methotrexate can be successfully used for cervical pregnancy treatment or transvaginal ultrasound-guided aspiration in combination with single-dose methotrexate administered systemically. Intra-lesional injection of the drug is preferable and more effective than systemic chemotherapy, but even this issue may still hold some controversy.

We did not try to preserve the intra-uterine pregnancy due to our patient's condition and treatment decision. Various types of conservative management to save an intra-uterine pregnancy have been attempted. Simple cervical embryo aspiration under transvaginal ultrasonography guidance can be used and delivery of a healthy baby has been successful [11]. However, using this method, another case report stated that an intra-uterine pregnancy was complicated by development of uterine varices at the cervical site, although the outcome was satisfactory [12]. Spontaneous abortion of intra-uterine pregnancy can occur after various methods of cervical pregnancy termination [13]. Transvaginal ultrasound-guided aspiration of cervical pregnancy and instillation of a hypertonic solution of sodium chloride can be a valuable treatment for heterotopic cervical pregnancy, and its application does not threaten the intra-uterine pregnancy [14]. Many reports of successful conservative management of cervical pregnancy recommend the use of a combination of methods. Systemic methotrexate therapy combined with hysteroscopic local endocervical resection of the heterotopic gestational sac could be considered the treatment of choice. A methotrexate pretreatment and the prophylactic placement of a high cervical cerclage before curettage has been shown to be effective [15]. Angiographic uterine artery embolization after unsuccessful methotrexate treatment, followed by vacuum evacuation and curettage of the cervical canal, is one approach.

## Conclusions

A combined intra-uterine and cervical pregnancy is a very rare but possible event, particularly after ART, with a high risk of life-threatening hemorrhage; the only option may be to perform a life saving hysterectomy, thereby representing a diagnostic and therapeutic challenge. Conservative management of HP is effective and safe, and made possible by the use of multiple conservative modalities. We recommend, if available, a conservative treatment of HP with a tamponade of cervical pregnancy by a Foley catheter balloon, slightly modified with a cerclage suture's fixation of the Foley catheter at the level of external cervical os, since it can be safely used without any major complication and combined with other conservative methods if required.

## Consent

Written informed consent was obtained from the patient for publication of this case report and accompanying images. A copy of the written consent is available for review by the Editor-in-Chief of this journal.

## Competing interests

The authors declare that they have no competing interests.

## Authors' contributions

HT and SA analyzed and treated the patient. BR and BU performed the ultrasound examination of the patient. HD and SV performed all the work concerning IVF and ET procedures. EII and MB collected all the data and were major contributors to writing the manuscript. All authors read and approved the final manuscript.
